# MHC1/LILRB1 axis as an innate immune checkpoint for cancer therapy

**DOI:** 10.3389/fimmu.2024.1421092

**Published:** 2024-06-07

**Authors:** Ziyi Hu, Qiaodong Zhang, Zehua He, Xiaojian Jia, Wencan Zhang, Xu Cao

**Affiliations:** ^1^ Shanghai Frontiers Science Center for Drug Target Identification and Delivery, and the Engineering Research Center of Cell and Therapeutic Antibody of the Ministry of Education, School of Pharmaceutical Sciences, National Key Laboratory of Innovative Immunotherapy, Shanghai Jiao Tong University, Shanghai, China; ^2^ Department of Addiction Medicine, Shenzhen Clinical Research Center for Mental Disorders, Shenzhen Kangning Hospital & Shenzhen Mental Health Center, Shenzhen, China; ^3^ Shanghai Key Laboratory of Veterinary Biotechnology, School of Agriculture and Biology, Shanghai Jiao Tong University, Shanghai, China

**Keywords:** MHC1, LILRB1, innate immunity, cancer immunotherapy, immune regulation

## Abstract

Immune checkpoint blockades (ICBs) have revolutionized cancer therapy through unleashing anti-tumor adaptive immunity. Despite that, they are usually effective only in a small subset of patients and relapse can occur in patients who initially respond to the treatment. Recent breakthroughs in this field have identified innate immune checkpoints harnessed by cancer cells to escape immunosurveillance from innate immunity. MHC1 appears to be such a molecule expressed on cancer cells which can transmit a negative signal to innate immune cells through interaction with leukocyte immunoglobulin like receptor B1 (LILRB1). The review aims to summarize the current understanding of MHC1/LILRB1 axis on mediating cancer immune evasion with an emphasis on the therapeutic potential to block this axis for cancer therapy. Nevertheless, one should note that this field is still in its infancy and more studies are warranted to further verify the effectiveness and safety in clinical as well as the potential to combine with existing immune checkpoints.

## 1 Introduction

Immune checkpoint blockades (ICBs) are the most advanced methods for cancer treatment (1, 2). Through harnessing adaptive immunity, ICBs lead to long-lasting anti-tumor responses for certain types of cancers even at advanced stages (3, 4). Inspired by this tremendous success, recent efforts in cancer immunology have also recognized immune surveillance mediated by innate immune system. The MHC1/LILRB1 axis represents such a critical facet of the intricate interplay between cancer cells and the innate immune system, offering a unique perspective in the field of cancer immunotherapy (5, 6).

MHC1 molecules are expressed on the cell surface of all nucleated cells and primarily present peptides derived from intracellular proteins, including both self-proteins and viral proteins (5). These peptides are typically 8 to 10 amino acids in length and are bound within a peptide-binding groove formed by the α1 and α2 domains of the MHC1 molecule (7). The binding specificity of MHC1 molecules is influenced by the amino acid residues within the peptide-binding groove, which interact with specific amino acids of the presented peptide. This interaction contributes to the stability of the peptide-MHC complex and determines the recognition by cytotoxic T lymphocytes (CTLs), leading to the mobilization of adaptive immunity (8). In the context of cancer immunology, orchestrating immune recognition of MHC1 molecules is responsible for presenting fragments of tumor-specific proteins (antigens) to CTLs which serves as the fundamental step for immune system to identify and eliminate cancer cells (9, 10).

Leukocyte immunoglobulin like receptor B1 (LILRB1), also known as ILT2 (Immunoglobulin-Like Transcript 2) or CD85j, is a cell surface receptor that belongs to the immunoglobulin superfamily (11, 12). It is primarily expressed on various innate immune cells, including natural killer (NK) cells, monocytes, macrophages, and dendritic cells (13). LILRB1 functions as an immune inhibitory receptor and is involved in immune regulation and modulation of immune responses (14). It interacts with specific ligands on target cells, which can be either classical or non-classical MHC1 molecules expressed on the cell surface. Through engaging MHC1, LILRB1 transmits inhibitory signals that dampen immune responses (6). As a result, activation of LILRB1 can impact the clearance of pathogens and/or tumor cells through inhibition of innate immune system. Correspondingly, disrupting MHC1/LILRB1 has shown both preclinical and clinical therapeutic activity against several types of cancers, behaving like the established immune checkpoints such as PD-1/PD-L1 and CD47/Sirpα which have been extensively reviewed elsewhere (15, 16).

This review focuses on this newly emerged immune checkpoint and summarizes the current understanding of MHC1/LILRB1 axis on mediating cancer evasion from innate immune cells and the therapeutic potential to block this axis for cancer therapy. Additionally, it proposes that further studies are warranted to verify the effectiveness and safety in clinical as well as the synergistic potential to combine with existing immune checkpoint blockades.

## 2 Structure and mechanism of MHC1/LILRB1 interaction

**Figure 1:**
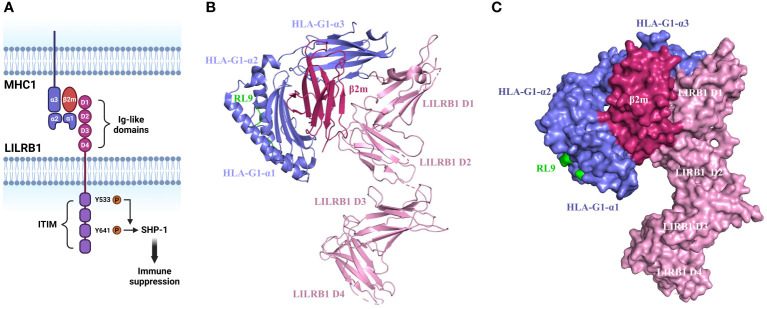
Structure and mechanism of MHC1/LILRB1 interaction. **(A)** LILRB1 contains four extracellular immunoglobulin (Ig)-like domains (D1-D4). The binding sites for HLA class I/β2-microglobulin (β2m) molecules localize to the apical D1-D2 region. The intracellular portion comprises four ITIMs. Critical tyrosine residues involved in the recruitment of SHP-1 within ITIM domains are indicated. **(B)** Cartoon structure of HLA-G1/β2m/RL9 complexed with LILRB1 (PDB ID: 6AEE). HLA-G1 is shown in purple, β2m is shown in rose red, RL9 is shown in green, and LILRB1 is shown in pink. This diagram was created with PyMOL software (20). **(C)** Surface diagram of the complex structure of HLA-G1/β2m/RL9 and LILRB1 (PDB ID: 6AEE). HLA-G1 is depicted in purple, β2m in rose red, RL9 in green, and LILRB1 in pink. This diagram was generated using PyMOL software (20).

MHC1 molecules consist of ternary complexes containing α chain (the MHC heavy chain), soluble serum protein β2 microglobulin (β2m), and bound peptide (7, 17). The α1 and α2 domains on the α chain form the peptide binding groove and contact the T-cell receptor, while α3 domain engages with the T cell co-receptor CD8 during T cell recognition. Differently, MHC1 binds to LILRB1 via α3 domain and β2m (18, 19). The β2m subunit is essential for the binding of MHC1 to the LILRB1 receptor, as evidence clearly shows the incapability of LILRB1 to recognize β2m-free forms of MHC1 molecules (6). On top of this, β2m is also necessary for the proper folding and stability of the MHC I molecule (**Figure 1**) (20).

The binding site on LILRB1 localizes on the two membrane-distal Ig domains while the other two Ig domains are not involved as disclosed by the crystal structure analysis (21). The same crystallographic study also reveals that LILRB1 interacts with the α3 domain of MHC1 via the N-terminal of D1 domain and the β2m subunit via the hinge region of D1-D2 domain (22). This engagement induces a conformational alternation of LILRB1 and thereby exposure of the intracellular phosphorylated immunoreceptor tyrosine-based inhibitory motif (ITIM) domain to Src family protein-tyrosine kinases (23). Upon the tyrosine residue is phosphorylated, the pITIM recruits Src homology 2 (SH2) domain containing phosphatases such as PTPN6, SHP-1, SHIP and SHP-2. For instance, the phosphorylation of Y533 has been shown to be involved in SHP-1 recruitment with Y614 as the main docking site (24–26). The activated phosphatase leads to the inactivation of ITAMs and inhibition of kinases critical for immune cell activation including SRC, PI3K/AKT, Syk/Zap70 and others (**Figure 1**) (27). For these reasons, LILRB1 is considered as an immunosuppressive receptor and its engagement with MHC1 leads to suppressive immune responses.

## 3 Regulatory effect of the MHC1/LILRB1 Axis on innate immune cells

LILRB1 is the most widely expressed LILRB family member and its expression is demonstrated on various immune cell populations, including monocytes/macrophages, dendritic cells, and subsets of NK cells and T cells (28, 29). Therefore, LILRB1 activation through engaging MHC1 molecules are likely involved in broad spectrum of immune regulation, particularly in innate immune cells considering its high abundance on these cells.

### 3.1 MHC1–LILRB1 signaling in macrophages

Macrophages, being major components in innate immune systems, exhibit notable plasticity and functional diversity (30). Being professional phagocytes and antigen presenting cells, they play a substantial role in the modulation of immune responses (31). Within the microenvironment of tumors, macrophages engage in interactions with cancer cells and are subject to regulation by a diverse repertoire of signaling molecules (32, 33). These intricate interactions and regulatory mechanisms exert a profound impact on the immune activity and function of macrophages.

The rapid advancements in cancer immunotherapy have underscored the critical need for fundamental investigations into immunoregulation across both adaptive and innate cell lineages (2). Tumor-capturing antibodies have demonstrated clinical utility in the treatment of various cancers, such as lymphomas, breast cancer, and colon cancer, etc (34–40). These antibodies exhibit diverse mechanisms of action, but their effectiveness is partially attributed to their capacity to induce antibody-dependent cellular phagocytosis of cancer cells (41). A recent study by Weissman’s group has highlighted that the expression of MHC1 on tumor cells can impede macrophage-mediated phagocytosis. Importantly, they have found that tumor cells can harness the upregulation of MHC1 molecules as a strategy to effectively inhibit macrophage-mediated phagocytosis, as indicated by the clear correlation of MHC1 expression level and resistance to macrophage phagocytosis. Intriguingly, removal of MHC1 molecules from cancer cells through CRISPR-mediated knockout clearly suppresses tumor growth *in vivo* at a macrophage-dependent manner, suggesting that MHC1 as a potential target for cancer immunotherapy (23).

Monocyte-lineage cells have been observed to express two MHC1-binding proteins namely LILRB1 and LILRB2, both of which not only bind to MHC1 but also possess ITIMs responsible for transmitting inhibitory signals intracellularly (42, 43). These characteristics position LILRB1 and LILRB2 as possible interacting partners on macrophages for mediating MHC1-associated suppression of phagocytosis. However, Barkal et al. has shown that LILRB1 but not LILRB2 is highly expressed in various subsets of tumor associated macrophages from human patients and interacts with MHC1 molecules, thereby exerting its inhibitory function on macrophage activity (23).This finding holds significant implications for understanding the immunoregulatory mechanisms of macrophages and developing more effective strategies for cancer immunotherapy. The study lays the groundwork for exploring LILRB1’s functions, regulatory mechanisms, and its potential in immunotherapy. The downregulation of MHC expression enhances the susceptibility of cancer cells to macrophage-mediated attack (44). A significant number of cancer types demonstrate compromised or absent surface MHC expression, which leads to impaired presentation of cancer neo-antigens (45). Consequently, patients bearing this type of tumors may not be suitable candidates for T cell-based therapies. However, intriguingly, these individuals could potentially represent ideal candidates for immunotherapies that harness the power of macrophages phagocytosis (46).

**Figure 2:**
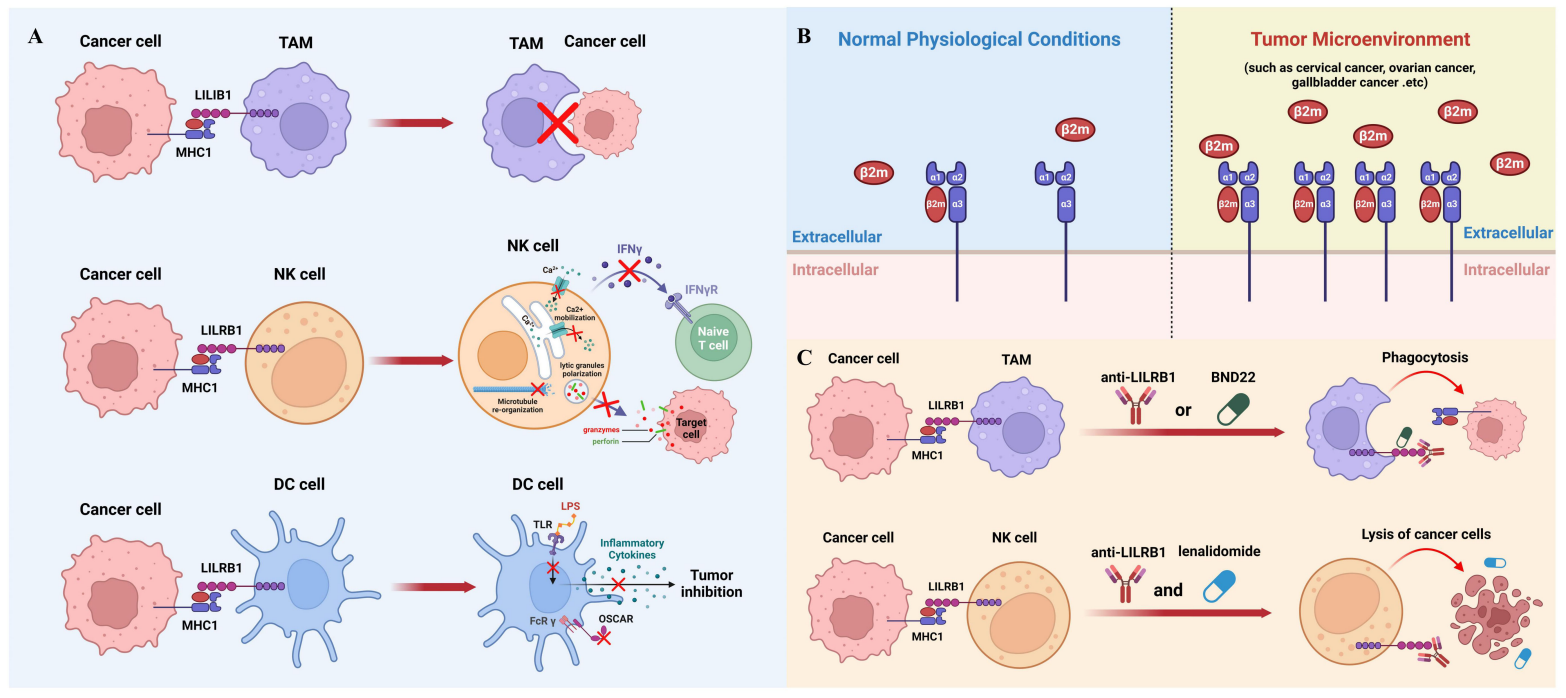
The role of MHC1/LILRB1 axis on innate immune cells and cancer therapy. **(A)** MHC1 on cancer cells transmits a negative signal through LILRB1 to inhibit the phagocytic ability of TAMs (top panel). NK cells express markedly high levels of LILRB1 molecules, which, upon engagement with MHC1 on tumor cells, leads to suppression of NK cell activity (middle panel). Cancer cell MHC1 induces tolerogenic DCs through LILRB1 (bottom panel). **(B)** The interaction between MHC1 and LILRB1 primarily involves the β2m subunit. β2m levels are typically low in serum and synovial fluid under normal conditions but are significantly upregulated in various cancers, including cervical, ovarian, and gallbladder cancer, serving as a significant prognostic marker. **(C)** Blocking the MHC1/LILRB1 axis with monoclonal antibody or targeted drug has shown remarkable anti-tumor activity through harnessing tumoricidal activity of macrophages and NK cells.

While macrophage-mediated immunotherapy has been extensively studied and has shown promising results in clinical practice (47), it remains an evolving field that requires further refinement. Novel immunotherapy strategies can be developed by effectively modulating the activity and function of macrophages like blocking MHC1/LILRB1 axis to suit different cancer types and individual patient characteristics (48). Nevertheless, there is still a need for ongoing research to optimize the efficacy of macrophage-mediated immunotherapy and overcome potential challenges and limitations associated with its implementation (**Figure 2A**).

### 3.2 MHC1–LILRB1 signaling in NK cells

NK cells play crucial roles in mediating anticancer immunity, and their effectiveness against cancer cells can be enhanced by engaging activating receptors or blocking inhibitory receptors (49, 50). Among the activating receptors, FcγRIII (CD16) is particularly important in antibody-dependent cellular cytotoxicity (ADCC) induced by therapeutic monoclonal antibodies (mAbs) (51, 52). These mAbs, such as rituximab for lymphoma, daratumumab for multiple myeloma (MM), cetuximab, and trastuzumab for metastatic solid cancer, engage CD16 and promote NK cell-mediated tumor cell destruction (53–58). Additionally, antibodies targeting the ligands of activating receptors like NKG2D and NKp46 have shown promising results in preclinical studies, indicating NK cell-dependent anti-tumor immunity (59, 60). Moreover, blocking inhibitory receptors on NK cells, such as LILRB1, KIR and NKG2A, with specific antibodies has been demonstrated to enhance NK cell function against cancer cells (61–65). Therefore, the function of NK cells is governed by a delicate balance between activating and inhibitory signals within the cell.

Unlike macrophages, the expression of LILRB1 is only restricted to a subpopulation of NK cells and at lower level (66). For example, the surface level of LILRB1 varies significantly on NK cells between healthy individuals (67). Usually, the LILRB1 level is relatively higher on FcγIIIA^+^CD^low^56 NK cells compared to FcγIIIA^-^CD^high^56 NK cells (68). While the clear reason for this differential expression remains obscure, it is likely that LILRB1 serves as a negative-feedback controlling mechanism to dampen FcγIIIA-dependent NK activation. Recent findings have shown that LILRB1 engagement by MHC1 reduces the activating synapse of NK cells via suppressing microtube re-organization and polarization of lytic granules (69). Meanwhile, the ligation also inhibits Ca^2+^ mobilization, the release of interferon γ and therefore impairs NK cell mediated cytotoxicity (24, 70).

It is worth mentioning that LILRB1 is not the sole receptor for MHC1 engagement on NK cells. Killer inhibitory receptors (KIR), which are ubiquitously expressed on all types of NK cells, are the main MHC1 binding partners on NK cells (71). Upon engagement with MHC1, KIR receptors transmit inhibitory signals to the cytolytic machinery of NK cells, thereby preventing the lysis of target cells (72). The inhibitory signal is mediated, in part, by the binding of the Src homology domain-containing tyrosine phosphatase (SHP-1) to ITIMs within the cytoplasmic domains of KIRs (73). This interaction leads to the dephosphorylation of signaling molecules that are crucial for the activation of NK cell cytolytic functions and release of inflammatory cytokines (**Figure 2A**).

### 3.3 MHC1–LILRB1 signaling in DC cells

Dendritic cells (DCs) are the most effective antigen presentation cells in the immune system, bridging the activation of innate and adaptive immunity (74). They are capable of sensing imbalances in the body, secreting cytokines and growth factors, and processing antigens for presentation to T helper (Th) cells, thereby inducing initial T cell activation and differentiation into effector cells (75). The precursor of DCs are monocytes or CD34^+^ progenitor cells which are highly expressed LILRB1. In differentiated DCs, the expression of LILRB1 is slightly upregulated and present in nearly all lineages upon maturation (13, 76). Additionally, stimulation with toll-like receptor (TLR) agonists such as lipopolysaccharide (LPS) or imiquimod has been demonstrated to further upregulate LILRB1 level in some subset of DCs, suggesting the dynamic expression of LILRB1 under various circumstances (13).

DCs have been implicated in the induction of immune tolerance through their interaction with MHC1-LILRB1. This interaction transmits inhibitory signals that contribute to the suppression of immune responses, thereby preventing excessive activation and promoting tolerance. For instance, in the presence of a LILRB1 ligand, DCs cannot be fully activated by LPS and are unable to secrete inflammatory cytokines (13, 28, 77). Moreover, the ligation of LILRB1 with MHC1 also compromises the immune-stimulating effect of the osteoclast-associated receptor which is an activating FcR γ- chain associated myeloid receptor involved in antigen presentation (78). Therefore, dysregulation of MHC1-LILRB1 signaling in DCs has been linked to the development of autoimmune disorders and cancer. Alterations in this pathway can have implications for immune surveillance, self-tolerance, and immune evasion by tumor cells (**Figure 2A**).

## 4 Emerging role of MHC1/LILRB1 axis as an innate immune checkpoint

Immune checkpoint proteins are regulatory molecules that act as gatekeepers of immune responses (79). These receptors usually contain intracellular ITIM or immunoreceptor tyrosine-based switch motif (ITSM) and use them to deliver suppressing signals to immune cells (80). In the past decades, several immune checkpoints, such as PD-1, CTLA4, LAG3, and TIM3, have been identified and usually associated with adaptive immunity, primarily regulating the function of T cells (79, 81–83).

In recent years, the concept of immune checkpoints has transformed cancer immunotherapy, allowing for remarkable advances in the field. In contrast, innate immune checkpoints are a relatively new concept, involving receptors and ligands regulating innate immune cell activity (84, 85). While much of the focus has been on adaptive immune checkpoints like PD-1/PD-L1, emerging research highlights the importance of innate immune checkpoints, with the MHC1/LILRB1 axis as the most recent one.

### 4.1 Role of MHC1/LILRB1 axis in cancer

MHC1 is a fundamental component of the immune system, responsible for presenting antigenic peptides to T cells (86). Although MHC1 is traditionally associated with adaptive immune recognition, its engagement in innate immunity is gaining increasing recognition (87, 88). LILRB1 is predominantly expressed on innate immune cells, such as macrophages and dendritic cells (13, 23). LILRB1 can interact with MHC1 on tumor cells, forming an axis to transmit negative signals to innate immune cells (23, 89). Therefore, when LILRB1 ligates MHC1, it inhibits innate immune cells mediated anti-tumor responses.

Numerous studies have revealed that cancer cells can actively exploit this axis to evade immune surveillance by the innate immune system. As mentioned above, the main subunit on MHC1 interacting with LILRB1 is β2m, which remains at a low level in serum and synovial fluid under normal physiological conditions. In contrast, the elevation of β2m has been shown to be a significant prognostic marker for various types of cancers such as cervical cancer, ovarian cancer, gallbladder cancer and others (**Figure 2B**) (90–93). Since β2m is a non-covalent binder to MHC1 molecules, upregulation of β2m could increase the level MHC1 complexes to interact with LILRB1 and therefore help cancer cells evade the innate immune system during tumor progression (94). However, the mechanisms underlying the dynamic regulation of β2m expression remain unexplored and warrant further investigations. It is worth mentioning that some cancer cells could reduce the expression of MHC1 molecules on their surface, because in this way they can hide themselves from the recognition and killing by cytotoxic T cells (95). This also makes LILRB1 a better target than MHC1 from a therapeutic point of view.

In Tumor microenvironment (TME), the expression of LILRB1 is mainly restricted in tumor stroma with tumor-associated macrophages as the major immune cell populations. This has been demonstrated in various types of cancers (96, 97). Importantly, further analysis has shown that LILRB1-expressing macrophages bears an M2- like phenotype, probably due to tumor cell mediated education through MHC1 molecules (98). Notably, it is reported that the ratio of LILRB1 expressing NK cells is higher in the peripheral blood of cancer patients compared to healthy controls (67). Recent studies have suggested that the abundance of LILRB1 in the TME could serve as a prognostic feature as well. For example, a clear association of LILRB1 expression level with advanced tumor stages and inferior survival rate has been demonstrated in gastric cancer (99). In ovarian cancer, a shorter survival, and worse adjuvant chemotherapy responses can be predicted by a high content of LILRB1-positive immune cells (100). The intricate relationship between LILRB1 expression and the tumor microenvironment highlights its potential as a significant prognostic marker across different cancers, providing valuable insights into disease progression and treatment outcomes.

### 4.2 Targeting MHC1/LILRB1 axis in cancer therapy

Due to the important inhibitory function of MHC1/LILRB1 axis on innate immune cells, therapeutic disruption of this interaction may offer beneficial effects through unleashing anti-tumor innate immunity. Barkal et al. initially used GHI/75, a monoclonal antibody derived from murine hybridoma, to block MHC1/LILRB1 interaction. They showed that this antibody enhances macrophage-mediated phagocytosis of solid tumor cells which can be largely boosted when combining with CD47-targeted antibody magrolimab. Additionally, LILRB1 antibody can increase CD20 antibody-induced phagocytosis and EGFR antibody-induced phagocytosis in the presence or absence of an Fc-silent CD47 antibody (23). Together, this compelling evidence suggests that LILRB1 blockade can induce macrophage-mediated tumor elimination through enhancing antibody-dependent cellular phagocytosis (**Figure 2C**).

Emerging evidence also shows that LILRB1 blockade leads to anti-tumor effects through NK cells. For example, in chronic lymphocytic leukimia, combination of LILRB1 antibody with lenalidomide promotes lysis of tumor cells through activation of NK cells (101). LILRB1 antibody produces similar effects against glioblastoma cells at a NK cell dependent manner. B1–176 is a humanized, Fc-engineered antibody to disrupt LILRB1 signaling and shows enhanced natural cytotoxicity against various tumor cell lines and *in vivo* xenografts (102). As mentioned previously, LILRB1 has been shown to inhibit antibody-mediated cellular cytotoxicity by NK cells (48). Thus, the combination of LILRB1 blockade with cetuximab has been tested in triple-negative breast cancer and showed significant synergistic effects in elimination of tumor cells through NK cells (**Figure 2C**) (103).

The above promising results in preclinical models have paved the way for clinical trials for LILRB1 antagonists in patients with cancer. BND-22 is a promising therapeutic agent that specifically targets the LILRB1 receptor. It is an antibody antagonist designed to disrupt the interactions between LILRB1 and its ligands, thereby modulating the immune response against solid tumors (104). Preclinical studies have demonstrated the potential of BND-22 in inhibiting tumor growth and metastasis. By blocking the LILRB1-mediated inhibitory signals, BND-22 promotes the activation of natural killer (NK) cells and CD8^+^ lymphocytes, which are key components of the antitumor immune response. This activation leads to enhanced cytotoxicity against tumor cells and improved tumor clearance. One of the mechanisms by which BND-22 exerts its antitumor effects is through the activation of macrophages. Normally, LILRB1 signaling in macrophages prevents them from engulfing tumor cells, allowing the tumor to evade immune surveillance. By blocking LILRB1 with BND-22, macrophages are relieved from this inhibitory signal and can effectively phagocytose tumor cells, leading to tumor regression. In preclinical models, BND-22 has shown promising results in various types of solid tumors, including melanoma and colorectal cancer (104). It has demonstrated significant inhibition of tumor growth, prolonged survival, and reduced metastasis. These findings suggest that targeting LILRB1 with BND-22 may have therapeutic potential in the treatment of solid tumors. Further studies are needed to evaluate the safety, efficacy, and optimal dosing strategies of BND-22 in clinical settings (104). Clinical trials are currently being conducted to assess the therapeutic benefits of BND-22 in patients with solid tumors (clinical trial identifier: NCT04717375). If successful, BND-22 could potentially become an important addition to cancer immunotherapy, providing a novel approach to enhance immune responses against solid tumors and improve patient outcomes (**Figure 2C**).

## 5 Perspectives

The cancer-immune axis serves as a crucial cellular checkpoint, by which the immune system controls immune responses against cancer. In contrast, cancer cells often hijack these mechanisms to evade immune surveillance and progress. MHC1/LILRB1 seems to be such an axis to regulate innate immune responses for various types of cancer. Intriguingly, therapeutic blockades disrupting MHC1/LILRB1 interaction have been advanced in clinical trials, displaying a great promise for the therapy of cancer. Nevertheless, one should note that this field is still in its infancy and more studies are warranted to further investigate research directions including but not limited to (1) identification of possible alternative ligands on cancer cells to interact with LILRB1; (2) assess the function of immune cells according to induvial patients upon LILRB1 blockade treatment; (3) evaluate alteration of cytokine profiles of immune cells when inhibiting LILRB1; (4) demonstrate to what extent the expression of other inhibitory receptors may impact the efficacy of LILRB1 targeting agents and (5) therefore discover the rational combination for synergism to test in preclinical and clinical studies. Ultimately, it is hopeful that completion of these studies will help turn this promising strategy into a real therapy for human cancer.

## Author contributions

ZHu: Writing – original draft, Writing – review & editing, Conceptualization, Investigation, Project administration, Visualization. QZ: Writing – original draft, Writing – review & editing, Software, Visualization. ZHe: Writing – original draft, Writing – review & editing, Visualization. XJ: Writing – original draft, Writing – review & editing, Investigation. WZ: Writing – original draft, Writing – review & editing, Investigation. XC: Writing – original draft, Writing – review & editing, Conceptualization, Funding acquisition, Investigation, Project administration, Supervision.

## References

[1] Gubin MM Zhang X Schuster H Caron E Ward JP Noguchi T. Checkpoint blockade cancer immunotherapy targets tumour-specific mutant antigens. Nature. (2014) 515:577–81. doi: 10.1038/nature13988 PMC4279952 25428507

[2] Sharma P Goswami S Raychaudhuri D Siddiqui BA Singh P Nagarajan A. Immune checkpoint therapy-current perspectives and future directions. Cell. (2023) 186:1652–69. doi: 10.1016/j.cell.2023.03.006 37059068

[3] Abdel-Hafiz HA Schafer JM Chen X Xiao T Gauntner TD Li Z. Y chromosome loss in cancer drives growth by evasion of adaptive immunity. Nature. (2023) 619:624–31. doi: 10.1038/s41586-023-06234-x PMC10975863 37344596

[4] Yan L Wu M Wang T Yuan H Zhang X Zhang H. Breast cancer stem cells secrete mif to mediate tumor metabolic reprogramming that drives immune evasion. Cancer Res. (2024) 84:1270–85. doi: 10.1158/0008-5472.CAN-23-2390 38335272

[5] Mohammed F Stones DH Willcox BE. Application of the immunoregulatory receptor lilrb1 as a crystallisation chaperone for human class I mhc complexes. J Immunol Methods. (2019) 464:47–56. doi: 10.1016/j.jim.2018.10.011 30365927

[6] Zhao J Zhong S Niu X Jiang J Zhang R Li Q. The mhc class I-lilrb1 signalling axis as a promising target in cancer therapy. Scand J Immunol. (2019) 90:e12804. doi: 10.1111/sji.12804 31267559

[7] Vetter W Rosenfelder N Kraan S Hiebl J. Structure and origin of the natural halogenated monoterpene mhc-1 and its concentrations in marine mammals and fish. Chemosphere. (2008) 73:7–13. doi: 10.1016/j.chemosphere.2008.06.020 18656231

[8] Pymm P Illing PT Ramarathinam SH O'Connor GM Hughes VA Hitchen C. Mhc-I peptides get out of the groove and enable a novel mechanism of hiv-1 escape. Nat Struct Mol Biol. (2017) 24:387–94. doi: 10.1038/nsmb.3381 PMC7900914 28218747

[9] Dong B Obermajer N Tsuji T Matsuzaki J Bonura C Withers H. Nk receptors replace cd28 as the dominant source of signal 2 for cognate recognition of cancer cells by taa-specific effector cd8(+) T cells. Res Sq. (2023). doi: 10.21203/rs.3.rs-3399211/v1

[10] Lei X de Groot DC Welters MJP de Wit T Schrama E van Eenennaam H. Cd4(+) T cells produce ifn-I to license cdc1s for induction of cytotoxic T-cell activity in human tumors. Cell Mol Immunol. (2024) 21:374–92. doi: 10.1038/s41423-024-01133-1 38383773 PMC10978876

[11] Banham AH Colonna M Cella M Micklem KJ Pulford K Willis AC. Identification of the cd85 antigen as ilt2, an inhibitory mhc class I receptor of the immunoglobulin superfamily. J Leukoc Biol. (1999) 65:841–5. doi: 10.1002/jlb.65.6.841 10380908

[12] Gustafson CE Qi Q Hutter-Saunders J Gupta S Jadhav R Newell E. Immune checkpoint function of cd85j in cd8 T cell differentiation and aging. Front Immunol. (2017) 8:692. doi: 10.3389/fimmu.2017.00692 28659925 PMC5469909

[13] Young NT Waller EC Patel R Roghanian A Austyn JM Trowsdale J. The inhibitory receptor lilrb1 modulates the differentiation and regulatory potential of human dendritic cells. Blood. (2008) 111:3090–6. doi: 10.1182/blood-2007-05-089771 18094328

[14] Lentz RW Colton MD Mitra SS Messersmith WA. Innate immune checkpoint inhibitors: the next breakthrough in medical oncology? Mol Cancer Ther. (2021) 20:961–74. doi: 10.1158/1535-7163.MCT-21-0041 PMC9028741 33850005

[15] Gordon SR Maute RL Dulken BW Hutter G George BM McCracken MN. Pd-1 expression by tumour-associated macrophages inhibits phagocytosis and tumour immunity. Nature. (2017) 545:495–9. doi: 10.1038/nature22396 PMC5931375 28514441

[16] Cao X Wang Y Zhang W Zhong X Gunes EG Dang J. Targeting macrophages for enhancing cd47 blockade-elicited lymphoma clearance and overcoming tumor-induced immunosuppression. Blood. (2022) 139:3290–302. doi: 10.1182/blood.2021013901 PMC9164740 35134139

[17] Winkler K Winter A Rueckert C Uchanska-Ziegler B Alexiev U. Natural mhc class I polymorphism controls the pathway of peptide dissociation from hla-B27 complexes. Biophys J. (2007) 93:2743–55. doi: 10.1529/biophysj.106.096602 PMC1989716 17573425

[18] Hassan I Ahmad F. Structural diversity of class I mhc-like molecules and its implications in binding specificities. Adv Protein Chem Struct Biol. (2011) 83:223–70. doi: 10.1016/B978-0-12-381262-9.00006-9 21570669

[19] Radaev S Rostro B Brooks AG Colonna M Sun PD. Conformational plasticity revealed by the cocrystal structure of nkg2d and its class I mhc-like ligand ulbp3. Immunity. (2001) 15:1039–49. doi: 10.1016/s1074-7613(01)00241-2 11754823

[20] Owen BA Pease LR. Thermal stability of mhc class I-beta 2-microglobulin peptide complexes in the endoplasmic reticulum is determined by the peptide occupancy of the transporter associated with antigen processing complex. J Immunol. (2001) 166:1740–7. doi: 10.4049/jimmunol.166.3.1740 11160219

[21] Wang Q Song H Cheng H Qi J Nam G Tan S. Structures of the four ig-like domain lilrb2 and the four-domain lilrb1 and hla-G1 complex. Cell Mol Immunol. (2020) 17:966–75. doi: 10.1038/s41423-019-0258-5 PMC7609294 31273318

[22] Willcox BE Thomas LM Bjorkman PJ. Crystal structure of hla-A2 bound to lir-1, a host and viral major histocompatibility complex receptor. Nat Immunol. (2003) 4:913–9. doi: 10.1038/ni961 12897781

[23] Barkal AA Weiskopf K Kao KS Gordon SR Rosental B Yiu YY. Engagement of mhc class I by the inhibitory receptor lilrb1 suppresses macrophages and is a target of cancer immunotherapy. Nat Immunol. (2018) 19:76–84. doi: 10.1038/s41590-017-0004-z 29180808 PMC5832354

[24] Colonna M Navarro F Bellon T Llano M Garcia P Samaridis J. A common inhibitory receptor for major histocompatibility complex class I molecules on human lymphoid and myelomonocytic cells. J Exp Med. (1997) 186:1809–18. doi: 10.1084/jem.186.11.1809 PMC2199153 9382880

[25] Bellon T Kitzig F Sayos J Lopez-Botet M. Mutational analysis of immunoreceptor tyrosine-based inhibition motifs of the ig-like transcript 2 (Cd85j) leukocyte receptor. J Immunol. (2002) 168:3351–9. doi: 10.4049/jimmunol.168.7.3351 11907092

[26] Sayos J Martinez-Barriocanal A Kitzig F Bellon T Lopez-Botet M. Recruitment of C-terminal src kinase by the leukocyte inhibitory receptor cd85j. Biochem Biophys Res Commun. (2004) 324:640–7. doi: 10.1016/j.bbrc.2004.09.097 15474475

[27] Kang X Kim J Deng M John S Chen H Wu G. Inhibitory leukocyte immunoglobulin-like receptors: immune checkpoint proteins and tumor sustaining factors. Cell Cycle. (2016) 15:25–40. doi: 10.1080/15384101.2015.1121324 26636629 PMC4825776

[28] Carenza C Calcaterra F Oriolo F Di Vito C Ubezio M Della Porta MG. Costimulatory molecules and immune checkpoints are differentially expressed on different subsets of dendritic cells. Front Immunol. (2019) 10:1325. doi: 10.3389/fimmu.2019.01325 31244860 PMC6579930

[29] Lewis Marffy AL McCarthy AJ. Leukocyte immunoglobulin-like receptors (Lilrs) on human neutrophils: modulators of infection and immunity. Front Immunol. (2020) 11:857. doi: 10.3389/fimmu.2020.00857 32477348 PMC7237751

[30] Weavers H Evans IR Martin P Wood W. Corpse engulfment generates a molecular memory that primes the macrophage inflammatory response. Cell. (2016) 165:1658–71. doi: 10.1016/j.cell.2016.04.049 PMC4912690 27212238

[31] Wang X Tokheim C Gu SS Wang B Tang Q Li Y. *In vivo* crispr screens identify the E3 ligase cop1 as a modulator of macrophage infiltration and cancer immunotherapy target. Cell. (2021) 184:5357–74 e22. doi: 10.1016/j.cell.2021.09.006 34582788 PMC9136996

[32] Kerzel T Giacca G Beretta S Bresesti C Notaro M Scotti GM. *In vivo* macrophage engineering reshapes the tumor microenvironment leading to eradication of liver metastases. Cancer Cell. (2023) 41:1892–910 e10. doi: 10.1016/j.ccell.2023.09.014 37863068

[33] Su X Liang C Chen R Duan S. Deciphering tumor microenvironment: cxcl9 and spp1 as crucial determinants of tumor-associated macrophage polarity and prognostic indicators. Mol Cancer. (2024) 23:13. doi: 10.1186/s12943-023-01931-7 38217023 PMC10790255

[34] Hsu YT Wu SJ Kao HW Hsiao SY Liao CK Chen TY. Glofitamab as a salvage treatment for B-cell lymphomas in the real world: A multicenter study in Taiwan. Cancer. (2024) 130:1972–81. doi: 10.1002/cncr.35217 38306242

[35] Sam J Hofer T Kuettel C Claus C Thom J Herter S. Cd19-cd28: an affinity-optimized cd28 agonist for combination with glofitamab (Cd20-tcb) as off-the-shelf immunotherapy. Blood. (2024) 143:2152–65. doi: 10.1182/blood.2023023381 38437725 PMC11143537

[36] Zhang Y Patel RP Kim KH Cho H Jo JC Jeong SH. Safety and Efficacy of a Novel Anti-Cd19 Chimeric Antigen Receptor T Cell Product Targeting a Membrane-Proximal Domain of Cd19 with Fast on- and Off-Rates against Non-Hodgkin Lymphoma: A First-in-Human Study. Mol Cancer. (2023) 22:200. doi: 10.1186/s12943-023-01886-9 38066564 PMC10709913

[37] Ramos-Martinez E Garcia-Vazquez FJ Falfan-Valencia R Rojas-Serrano J Alfaro-Cruz A Perez-Villasenor M. The type 2 inflammatory response favors recognition of tumor antigens by ige in breast cancer. Cancer Rep (Hoboken). (2024) 7:e2002. doi: 10.1002/cnr2.2002 38389406 PMC10884619

[38] Silvestri VL Tran AD Chung M Chung N Gril B Robinson C. Distinct uptake and elimination profiles for trastuzumab, human igg and biocytin-tmr in experimental her2+ Brain metastases of breast cancer. Neuro Oncol. (2024). doi: 10.1093/neuonc/noae025 PMC11145443 38363979

[39] DiPeri TP Evans KW Raso MG Zhao M Rizvi YQ Zheng X. Adavosertib enhances antitumor activity of trastuzumab deruxtecan in her2-expressing cancers. Clin Cancer Res. (2023) 29:4385–98. doi: 10.1158/1078-0432.CCR-23-0103 PMC10618648 37279095

[40] Zheng Y Zhou R Cai J Yang N Wen Z Zhang Z. Matrix stiffness triggers lipid metabolic cross-talk between tumor and stromal cells to mediate bevacizumab resistance in colorectal cancer liver metastases. Cancer Res. (2023) 83:3577–92. doi: 10.1158/0008-5472.CAN-23-0025 PMC10618741 37610655

[41] Cao X Chen J Li B Dang J Zhang W Zhong X. Promoting antibody-dependent cellular phagocytosis for effective macrophage-based cancer immunotherapy. Sci Adv. (2022) 8:eabl9171. doi: 10.1126/sciadv.abl9171 35302839 PMC8932662

[42] Mori Y Tsuji S Inui M Sakamoto Y Endo S Ito Y. Inhibitory immunoglobulin-like receptors lilrb and pir-B negatively regulate osteoclast development. J Immunol. (2008) 181:4742–51. doi: 10.4049/jimmunol.181.7.4742 18802077

[43] Pockley AG Vaupel P Multhoff G. Nk cell-based therapeutics for lung cancer. Expert Opin Biol Ther. (2020) 20:23–33. doi: 10.1080/14712598.2020.1688298 31714156

[44] Zheng N Wang T Luo Q Liu Y Yang J Zhou Y. M2 macrophage-derived exosomes suppress tumor intrinsic immunogenicity to confer immunotherapy resistance. Oncoimmunology. (2023) 12:2210959. doi: 10.1080/2162402X.2023.2210959 37197441 PMC10184604

[45] Bosch GJ Joosten AM Kessler JH Melief CJ Leeksma OC. Recognition of bcr-abl positive leukemic blasts by human cd4+ T cells elicited by primary in vitro immunization with a bcr-abl breakpoint peptide. Blood. (1996) 88:3522–7. doi: 10.1182/blood.V88.9.3522.bloodjournal8893522 8896419

[46] Antonsen KW Friis HN Sorensen BS Etzerodt A Moestrup SK Moller HJ. Comparison of culture media reveals that non-essential amino acids strongly affect the phenotype of human monocyte-derived macrophages. Immunology. (2023) 170:344–58. doi: 10.1111/imm.13670 37291897

[47] Dong H Qi Y Kong X Wang Z Fang Y Wang J. Pd-1/pd-L1 inhibitor-associated myocarditis: epidemiology, characteristics, diagnosis, treatment, and potential mechanism. Front Pharmacol. (2022) 13:835510. doi: 10.3389/fphar.2022.835510 35517794 PMC9062035

[48] Zeller T Munnich IA Windisch R Hilger P Schewe DM Humpe A. Perspectives of targeting lilrb1 in innate and adaptive immune checkpoint therapy of cancer. Front Immunol. (2023) 14:1240275. doi: 10.3389/fimmu.2023.1240275 37781391 PMC10533923

[49] Vivier E Nunes JA Vely F. Natural killer cell signaling pathways. Science. (2004) 306:1517–9. doi: 10.1126/science.1103478 15567854

[50] Waggoner SN Cornberg M Selin LK Welsh RM. Natural killer cells act as rheostats modulating antiviral T cells. Nature. (2011) 481:394–8. doi: 10.1038/nature10624 PMC3539796 22101430

[51] Yu X Menard M Prechl J Bhakta V Sheffield WP Lazarus AH. Monovalent fc receptor blockade by an anti-fcgamma receptor/albumin fusion protein ameliorates murine itp with abrogated toxicity. Blood. (2016) 127:132–8. doi: 10.1182/blood-2015-08-664656 26500340

[52] Green SK Karlsson MC Ravetch JV Kerbel RS. Disruption of cell-cell adhesion enhances antibody-dependent cellular cytotoxicity: implications for antibody-based therapeutics of cancer. Cancer Res. (2002) 62:6891–900. 12460904

[53] Cho J. Basic immunohistochemistry for lymphoma diagnosis. Blood Res. (2022) 57:55–61. doi: 10.5045/br.2022.2022037 35483927 PMC9057666

[54] Arora J Ayyappan SR Yin C Smith BJ Lemke-Miltner CD Wang Z. T cell help in the tumor microenvironment enhances rituximab-mediated nk cell adcc. Blood. (2024) 143:1816–24. doi: 10.1182/blood.2023023370 38457360 PMC11076912

[55] Liu J Xing L Li J Wen K Liu N Liu Y. Epigenetic regulation of cd38/cd48 by kdm6a mediates nk cell response in multiple myeloma. Nat Commun. (2024) 15:1367. doi: 10.1038/s41467-024-45561-z 38355622 PMC10866908

[56] Kassem S Diallo BK El-Murr N Carrie N Tang A Fournier A. Sar442085, a novel anti-cd38 antibody with enhanced antitumor activity against multiple myeloma. Blood. (2022) 139:1160–76. doi: 10.1182/blood.2021012448 35201323

[57] Veluchamy JP Spanholtz J Tordoir M Thijssen VL Heideman DA Verheul HM. Combination of nk cells and cetuximab to enhance anti-tumor responses in ras mutant metastatic colorectal cancer. PloS One. (2016) 11:e0157830. doi: 10.1371/journal.pone.0157830 27314237 PMC4912059

[58] Lee SC Shimasaki N Lim JSJ Wong A Yadav K Yong WP. Phase I trial of expanded, activated autologous nk-cell infusions with trastuzumab in patients with her2-positive cancers. Clin Cancer Res. (2020) 26:4494–502. doi: 10.1158/1078-0432.CCR-20-0768 32522887

[59] Khaleafi R Zeleznjak J Cordela S Drucker S Rovis TL Jonjic S. Reovirus infection of tumor cells reduces the expression of nkg2d ligands, leading to impaired nk-cell cytotoxicity and functionality. Front Immunol. (2023) 14:1231782. doi: 10.3389/fimmu.2023.1231782 37753084 PMC10518469

[60] Sen Santara S Lee DJ Crespo A Hu JJ Walker C Ma X. The nk cell receptor nkp46 recognizes ecto-calreticulin on er-stressed cells. Nature. (2023) 616:348–56. doi: 10.1038/s41586-023-05912-0 PMC10165876 37020026

[61] Trefny MP Rothschild SI Uhlenbrock F Rieder D Kasenda B Stanczak MA. A variant of a killer cell immunoglobulin-like receptor is associated with resistance to pd-1 blockade in lung cancer. Clin Cancer Res. (2019) 25:3026–34. doi: 10.1158/1078-0432.CCR-18-3041 30765392

[62] Handgretinger R Lang P Andre MC. Exploitation of natural killer cells for the treatment of acute leukemia. Blood. (2016) 127:3341–9. doi: 10.1182/blood-2015-12-629055 27207791

[63] Nguyen S Dhedin N Vernant JP Kuentz M Al Jijakli A Rouas-Freiss N. Nk-cell reconstitution after haploidentical hematopoietic stem-cell transplantations: immaturity of nk cells and inhibitory effect of nkg2a override gvl effect. Blood. (2005) 105:4135–42. doi: 10.1182/blood-2004-10-4113 15687235

[64] Haanen JB Cerundolo V. Nkg2a, a new kid on the immune checkpoint block. Cell. (2018) 175:1720–2. doi: 10.1016/j.cell.2018.11.048 30550781

[65] Carrette F Vivier E. Nkg2a blocks the anti-metastatic functions of natural killer cells. Cancer Cell. (2023) 41:232–4. doi: 10.1016/j.ccell.2023.01.008 36787695

[66] Lozano E Diaz T Mena MP Sune G Calvo X Calderon M. Loss of the immune checkpoint cd85j/lilrb1 on Malignant plasma cells contributes to immune escape in multiple myeloma. J Immunol. (2018) 200:2581–91. doi: 10.4049/jimmunol.1701622 29531171

[67] Fan J Li J Han J Zhang Y Gu A Song F. Expression of leukocyte immunoglobulin-like receptor subfamily B expression on immune cells in hepatocellular carcinoma. Mol Immunol. (2021) 136:82–97. doi: 10.1016/j.molimm.2021.05.011 34098344

[68] Ty M Sun S Callaway PC Rek J Press KD van der Ploeg K. Malaria-driven expansion of adaptive-like functional cd56-negative nk cells correlates with clinical immunity to malaria. Sci Transl Med. (2023) 15:eadd9012. doi: 10.1126/scitranslmed.add9012 36696483 PMC9976268

[69] Favier B Lemaoult J Lesport E Carosella ED. Ilt2/Hla-G Interaction Impairs Nk-Cell Functions through the Inhibition of the Late but Not the Early Events of the Nk-Cell Activating Synapse. FASEB J. (2010) 24:689–99. doi: 10.1096/fj.09-135194 19841038

[70] Morel E Bellon T. Hla class I molecules regulate ifn-gamma production induced in nk cells by target cells, viral products, or immature dendritic cells through the inhibitory receptor ilt2/cd85j. J Immunol. (2008) 181:2368–81. doi: 10.4049/jimmunol.181.4.2368 18684926

[71] Walter L Petersen B. Diversification of both kir and nkg2 natural killer cell receptor genes in macaques - implications for highly complex mhc-dependent regulation of natural killer cells. Immunology. (2017) 150:139–45. doi: 10.1111/imm.12666 PMC5215213 27565739

[72] Dulberger CL McMurtrey CP Holzemer A Neu KE Liu V Steinbach AM. Human leukocyte antigen F presents peptides and regulates immunity through interactions with nk cell receptors. Immunity. (2017) 46:1018–29 e7. doi: 10.1016/j.immuni.2017.06.002 28636952 PMC5523829

[73] Binstadt BA Brumbaugh KM Dick CJ Scharenberg AM Williams BL Colonna M. Sequential involvement of lck and shp-1 with mhc-recognizing receptors on nk cells inhibits fcr-initiated tyrosine kinase activation. Immunity. (1996) 5:629–38. doi: 10.1016/s1074-7613(00)80276-9 8986721

[74] Krimpenfort LT Degn SE Heesters BA. The follicular dendritic cell: at the germinal center of autoimmunity? Cell Rep. (2024) 43:113869. doi: 10.1016/j.celrep.2024.113869 38431843

[75] Binnewies M Mujal AM Pollack JL Combes AJ Hardison EA Barry KC. Unleashing type-2 dendritic cells to drive protective antitumor cd4(+) T cell immunity. Cell. (2019) 177:556–71 e16. doi: 10.1016/j.cell.2019.02.005 30955881 PMC6954108

[76] Corrigall VM Vittecoq O Panayi GS. Binding immunoglobulin protein-treated peripheral blood monocyte-derived dendritic cells are refractory to maturation and induce regulatory T-cell development. Immunology. (2009) 128:218–26. doi: 10.1111/j.1365-2567.2009.03103.x PMC2767311 19740378

[77] Gros F Cabillic F Toutirais O Maux AL Sebti Y Amiot L. Soluble hla-G molecules impair natural killer/dendritic cell crosstalk *via* inhibition of dendritic cells. Eur J Immunol. (2008) 38:742–9. doi: 10.1002/eji.200736918 18266268

[78] Besse B Charrier M Lapierre V Dansin E Lantz O Planchard D. Dendritic cell-derived exosomes as maintenance immunotherapy after first line chemotherapy in nsclc. Oncoimmunology. (2016) 5:e1071008. doi: 10.1080/2162402X.2015.1071008 27141373 PMC4839329

[79] Li X Wang X Ito A Tsuji NM. A nanoscale metal organic frameworks-based vaccine synergises with pd-1 blockade to potentiate anti-tumour immunity. Nat Commun. (2020) 11:3858. doi: 10.1038/s41467-020-17637-z 32737343 PMC7395732

[80] Fernandes RA Su L Nishiga Y Ren J Bhuiyan AM Cheng N. Immune receptor inhibition through enforced phosphatase recruitment. Nature. (2020) 586:779–84. doi: 10.1038/s41586-020-2851-2 PMC7875542 33087934

[81] Baldanzi G. Immune checkpoint receptors signaling in T cells. Int J Mol Sci. (2022) 23:3529. doi: 10.3390/ijms23073529 35408889 PMC8999077

[82] Arimura K Hiroshima K Nagashima Y Nakazawa T Ogihara A Orimo M. Lag3 is an independent prognostic biomarker and potential target for immune checkpoint inhibitors in Malignant pleural mesothelioma: A retrospective study. BMC Cancer. (2023) 23:1206. doi: 10.1186/s12885-023-11636-1 38062416 PMC10704683

[83] Joller N Anderson AC Kuchroo VK. Lag-3, tim-3, and tigit: distinct functions in immune regulation. Immunity. (2024) 57:206–22. doi: 10.1016/j.immuni.2024.01.010 PMC10919259 38354701

[84] Vivier E Rebuffet L Narni-Mancinelli E Cornen S Igarashi RY Fantin VR. Natural killer cell therapies. Nature. (2024) 626:727–36. doi: 10.1038/s41586-023-06945-1 38383621

[85] Zhang S Guo L Zhang Z Liu X Chen W Wei Y. Type-I protein arginine methyltransferase inhibition primes anti-Programmed cell death protein 1 immunotherapy in triple-Negative breast cancer. Cancer. (2023) 130:1415–23. doi: 10.1002/cncr.35142 38079306

[86] Wu X Li T Jiang R Yang X Guo H Yang R. Targeting mhc-I molecules for cancer: function, mechanism, and therapeutic prospects. Mol Cancer. (2023) 22:194. doi: 10.1186/s12943-023-01899-4 38041084 PMC10693139

[87] Ekeruche-Makinde J Miles JJ van den Berg HA Skowera A Cole DK Dolton G. Peptide length determines the outcome of tcr/peptide-mhci engagement. Blood. (2013) 121:1112–23. doi: 10.1182/blood-2012-06-437202 PMC3653566 23255554

[88] Dai H Lan P Zhao D Abou-Daya K Liu W Chen W. Pirs mediate innate myeloid cell memory to nonself mhc molecules. Science. (2020) 368:1122–7. doi: 10.1126/science.aax4040 PMC7379379 32381589

[89] Harrison TE Morch AM Felce JH Sakoguchi A Reid AJ Arase H. Structural basis for rifin-mediated activation of lilrb1 in malaria. Nature. (2020) 587:309–12. doi: 10.1038/s41586-020-2530-3 PMC7116854 32650338

[90] Bataille R Grenier J Sany J. Beta-2-microglobulin in myeloma: optimal use for staging, prognosis, and treatment–a prospective study of 160 patients. Blood. (1984) 63:468–76. doi: 10.1182/blood.V63.2.468.bloodjournal632468 6362753

[91] Stasi R Brunetti M Parma A Di Giulio C Terzoli E Pagano A. The prognostic value of soluble interleukin-6 receptor in patients with multiple myeloma. Cancer. (1998) 82:1860–6. doi: 10.1002/(SICI)1097-0142(19980515)82:10<1860::AID-CNCR7>3.3.CO;2-0 9587117

[92] Gujar S Dielschneider R Clements D Helson E Shmulevitz M Marcato P. Multifaceted therapeutic targeting of ovarian peritoneal carcinomatosis through virus-induced immunomodulation. Mol Ther. (2013) 21:338–47. doi: 10.1038/mt.2012.228 PMC3594021 23299799

[93] Sun J Yang ZL Miao X Zou Q Li J Liang L. Atp5b and beta2-microglobulin are predictive markers for the prognosis of patients with gallbladder cancer. J Mol Histol. (2015) 46:57–65. doi: 10.1007/s10735-014-9597-9 25311765

[94] Dirscherl C Lochte S Hein Z Kopicki JD Harders AR Linden N. Dissociation of beta2m from mhc class I triggers formation of noncovalent transient heavy chain dimers. J Cell Sci. (2022) 135:jcs259489. doi: 10.1242/jcs.259498 35393611

[95] Chen X Lu Q Zhou H Liu J Nadorp B Lasry A. A membrane-associated mhc-I inhibitory axis for cancer immune evasion. Cell. (2023) 186:3903–20.e21. doi: 10.1016/j.cell.2023.07.016 37557169 PMC10961051

[96] Zheng Y Chen Z Han Y Han L Zou X Zhou B. Immune suppressive landscape in the human esophageal squamous cell carcinoma microenvironment. Nat Commun. (2020) 11:6268. doi: 10.1038/s41467-020-20019-0 33293583 PMC7722722

[97] Li S Yu J Huber A Kryczek I Wang Z Jiang L. Metabolism drives macrophage heterogeneity in the tumor microenvironment. Cell Rep. (2022) 39:110609. doi: 10.1016/j.celrep.2022.110609 35385733 PMC9052943

[98] Nunez SY Ziblat A Secchiari F Torres NI Sierra JM Raffo Iraolagoitia XL. Human M2 macrophages limit nk cell effector functions through secretion of tgf-beta and engagement of cd85j. J Immunol. (2018) 200:1008–15. doi: 10.4049/jimmunol.1700737 29282306

[99] Zhang Y Wang H Xu X Liu H Hao T Yin S. Poor prognosis and therapeutic responses in lilrb1-expressing M2 macrophages-enriched gastric cancer patients. Front Oncol. (2021) 11:668707. doi: 10.3389/fonc.2021.668707 34485116 PMC8415088

[100] Xu X Yin S Wang Y Zhu Q Zheng G Lu Y. Lilrb1(+) immune cell infiltration identifies immunosuppressive microenvironment and dismal outcomes of patients with ovarian cancer. Int Immunopharmacol. (2023) 119:110162. doi: 10.1016/j.intimp.2023.110162 37075669

[101] Villa-Alvarez M Sordo-Bahamonde C Lorenzo-Herrero S Gonzalez-Rodriguez AP Payer AR Gonzalez-Garcia E. Ig-like transcript 2 (Ilt2) blockade and lenalidomide restore nk cell function in chronic lymphocytic leukemia. Front Immunol. (2018) 9:2917. doi: 10.3389/fimmu.2018.02917 30619281 PMC6297751

[102] Chen H Chen Y Deng M John S Gui X Kansagra A. Antagonistic anti-lilrb1 monoclonal antibody regulates antitumor functions of natural killer cells. J Immunother Cancer. (2020) 8:e000515. doi: 10.1136/jitc-2019-000515 32771992 PMC7418854

[103] Roberti MP Julia EP Rocca YS Amat M Bravo AI Loza J. Overexpression of cd85j in tnbc patients inhibits cetuximab-mediated nk-cell adcc but can be restored with cd85j functional blockade. Eur J Immunol. (2015) 45:1560–9. doi: 10.1002/eji.201445353 25726929

[104] Mandel I Haves Ziv D Goldshtein I Peretz T Alishekevitz D Fridman Dror A. Bnd-22, a first-in-class humanized ilt2-blocking antibody, promotes antitumor immunity and tumor regression. J Immunother Cancer. (2022) 10:e004859. doi: 10.1136/jitc-2022-004859 36096532 PMC9472153

